# Supplementary material for: Identifying fetal yawns based on temporal dynamics of mouth openings: A preterm neonate model using support vector machines (SVMs)
Source: PLoS One. 2019 Dec 19;14(12):e0226921. doi: 10.1371/journal.pone.0226921 (PMC6922391; doi:10.1371/journal.pone.0226921)
Supplement: Attachment — Submitted filename: Response to Reviewers.docx [file pone.0226921.s002.docx]

**Reviewer #1**

Q1: The neonates in this study were placed in a convenient position for observation during data collection, but the fetus will not be so co-operative. Moreover, the yawn of the near term fetus might occasionally be mechanically constrained because of its position. Further, the neonate is yawning in air, while the fetus will be immersed in amniotic fluid. The timings and definitions may need to be adjusted.

**RESPONSE:** We thank the reviewer for their appreciation of our work, and we agree with their remarks. However, as we argued for in a newly inserted paragraph in the general discussion (lines 455-478), the mentioned differences between fetuses and newborns should not hinder the applicability of the proposed method to fetuses, especially as yawn is universally recognized as a stereotyped behavioral pattern. Here is the new paragraph addressing the reviewer’s remarks:

“In order to establish this method as a viable option for identifying fetal yawns in clinical and research settings, additional work should be done to test it on fetal behavior. In particular, the suboptimal framing and variable quality that characterize ultrasonographic scans represent a ubiquitous issue in the study of fetal behavior. However, the fact that the proposed method only relies on the timing of mouth openings highlights a key advantage of this approach, that was specifically conceived in order to overcome the issues of identifying a complex facial behavior such as yawn from ultrasonographic scans. Another potential reason for concern regards the different conditions of fetuses compared with preterm neonates; for example, fetuses are immersed in amniotic fluid and the yawn of the near-term fetus might occasionally be mechanically constrained because of its position. However, there is unanimous agreement in the literature on the stability of the yawning behavioral pattern throughout life [12, 17, 21, 22, 23, 24]. Therefore, the advantages of using preterm neonates as a model for training classificatory algorithms far outweigh these potential issues.

A strength of the current study was the use of the SCPB [25], a rigorous coding system based on Baby FACS [22], to identify yawns. Because Baby FACS coding is based on multiple and redundant cues to each facial action, facial muscle actions can be reliably identified in fetuses [36], preterm and full-term neonates [37], and infants with facial anomalies [38] as well as typically developing infants. And because the basic coding units of Baby FACS are exhaustive and mutually exclusive, any complex facial movement can be precisely and unambiguously identified in terms of combinations and sequences of its constituent facial muscle actions. Therefore, Baby FACS, which has been referred to as the “gold standard” for coding infants’ facial expressions [39], is especially suited for coding yawning, sensory and perceptual responses [37], pain [40], and other fetal, neonatal and infant behaviors that don’t fit simplified templates for a limited set of emotional expressions.”

Q2: The paper would benefit from more data, sufficient to create an independent test set of equal size. The observation that your Model B is the best performing may be a statistical artifact, or a bit of over training, or it may be real. I can't say.

**RESPONSE:** We fully embrace the reviewer's suggestion and inserted this consideration as a new sentence (lines 441-443, reported below).

“Further research is needed in order to confirm these results on bigger samples, e.g. regarding the best performing model, as well as to provide additional training for the SVM algorithm.”

**Reviewer #2**

Q1: In the title it is probably better to replace “preterm model” with “preliminary model” or “initial model”. Unless the adjective “preterm” refers to neonates.

**RESPONSE:** We thank the reviewer for their appreciation of our work. Preterm indeed refers to neonates: To make it clear we changed the titles as follows: "Identifying fetal yawns based on temporal dynamics of mouth openings: A preterm neonate model using support vector machines (SVMs)".

Q2: How were SVM parameter values determined (C=100, gamma=1, line 316) and what does "type 1 error" and "type 2 error" (lines 313-314) mean?

**RESPONSE:** SVM parameters were set at the default value for the package adopted. Since results were optimal, and the aim of the Study 2 was to test the feasibility of the proposed SVM-based method, they were reported only for replicability purposes. Type I and II errors mean respectively false positives and negatives. A specification has been introduced (lines 326-327)

Q3: It would be worth describing more precisely how sensitivity and specificity were determined in study 1.

**RESPONSE:** The methods adopted to calculate sensitivity and specificity were explicitly reported at their first occurrence (lines 237-242, reported below).

“In order to assess the accuracy of the measure adopted by Reissland et al. [10], we used a contingency table to assess its sensitivity (i.e. the true positive rate, calculated as the proportion of yawns that were correctly classified as yawns) and specificity (i.e. the true negative rate, calculated as the proportion of non-yawns that were correctly classified as non-yawns), and compute Cohen’s Kappa.”

Q 4: The abbreviation "AU" is explained later (line 190) than its first use in the text (line 156).

**RESPONSE:** The "AU" abbreviation was explained at its first occurrence and all abbreviations were checked throughout the manuscript (line 129).

5) What does "t(126)" (lines 346-353) mean?

**RESPONSE:** The t-statistic is the ratio of the departure of the estimated value of a parameter from its hypothesized value to its standard error. The value in parentheses represents degrees of freedom.

Q6: English can be slightly improved in some places, e.g.:

Q6.1: “construct validity of the measure” -> “validity of the measure” or “correctness of the measure”

**RESPONSE:** Construct validity, defined as the degree to which a test measures what it claims, or purports, to be measuring, is one of the key concepts in psychometrics. Because this is technical terminology, we maintained "construct validity of the measure" in the manuscript, after consulting the English mother tongue author.

Q6.2: “risk of sacrificing accuracy for precision” -> “risk of lowering accuracy at the expense of precision” (line 96)

**RESPONSE:** In our understanding, the reviewer’s proposed sentence does not retain the original meaning, as precision is not affected. In order to make the phrase more immediately understandable to the reader and not redundant, we deleted "for precision" from the original formulation. (line 99)

Q6.3- line 385: “having achieved the highest” -> “having the highest” or “achieving the highest”

**RESPONSE:** In our understanding, the original wording correctly uses the “perfect participle” tense. “The perfect participle indicates completed action and was therefore maintained in the revised manuscript. (https://www.learnenglish.de/grammar/participleperfect.html)

Q6.4: line 389: “did not just display a longer” -> “not only showed a longer”

**RESPONSE:** The suggested version was adopted (line (403)

Q6.5: line 394: “according to one specific iteration” -> “in a given iteration”

**RESPONSE:** The original wording refers to a specific iteration taken at random, not to any iteration. However, Figure 2 was deleted from the new version of the manuscript because of its limited informative value.

- line 156: “et al” -> “et al.”, “F For” -> “For”, line 383: “011” -> “0.11”

**RESPONSE:** The suggested revisions were adopted.

**Reviewer #3**

Q1: The abbreviation usage needs improving. Each abbreviation shall be explained at the first use. The “symbol” t(126) (page 10) needs explanation as well?

**RESPONSE:** We thank the reviewer for their appreciation of our work. Abbreviations were checked and explained at their first use. The t-statistic is the ratio of the departure of the estimated value of a parameter from its hypothesized value to its standard error. The value in parentheses represents degrees of freedom. We propose to maintain the original, more concise formulation.

Q2: There are some typos, e.g.: “F For” -> “For” (line 156), “011” -> “0.11” (line 383). The text should be checked thoroughly.

**RESPONSE:** The manuscript was checked for typos and corrected.

Q3: How were SVM parameter values (C and γ) determined?

**RESPONSE:** SVM parameters were set at the default value for the package adopted. Since results were optimal, and the aim of Study 2 was to test the feasibility of the proposed SVM-based method, they were reported only for replicability purposes.

Q4: Is the classification performance defined on the testing sets only? If yes, the testing set would contain 5 yawing cases (on average). As the generalization ability estimation is of the crucial importance, is this number enough for making the conclusions about fetal yawn models?

**RESPONSE:** Classification performance was calculated based on testing sets only, but the hold-out validation included 30 iterations, and for each different training and testing sets were randomly selected. Moreover, both according to previous literature and our results, yawns seem to represent a very specific population of mouth openings, in terms of temporal dynamics. Therefore, we believe that the proposed sample is sufficient to show the feasibility of such method.

Q5: It seems that the figure 2 shows the SVM classification result for Model A (which is not explicitly stated). But what is its purpose? Why plateau duration was fixed at 1.05 s. (half of the mean of plateau duration related to fetal yawns)?

**RESPONSE:** The purpose of figure 2 was simply to illustrate how SVM models work in establishing hyper-planes for classifying mouth openings. Plateau duration was fixed around the shortest duration coded for yawns, in order to show more informative decision boundaries. The displayed model is Model D, because the adopted R package (e1071) did not allow for graphical representation of interaction-only models. In consideration of these limitations, and of its limited informative content, we propose to delete the figure from the new version of the manuscript.

Q6 As the classification results are so high, did Authors consider to model the fetal yawns by using only two temporal dynamics variables of the highest discrimination ability (like opening and plateau phases – see Figure 1)?

**RESPONSE:** Other methods were tried out in a preliminary phase. However, the difference between opening and closing (opening/closing asymmetry) also had high discrimination ability, and is directly related to the description, ubiquitous in the pertinent literature, of yawning as characterized by a long opening and a short closing phase.

**Reviewer #4**

Q1: Introduction. Regarding the difference among reported yawns frequencies, which is the role of circadian changes in yawns activity of the fetus. Does yawns activity change over time? I would suggest discussing this point.

**RESPONSE:** We thank the reviewer for their appreciation of our work and the valuable comments and suggestions. We inserted a new paragraph in the introduction (lines 54-59) in order to briefly discuss the potential effects of circadian rhythms and other variables (see below).

“The sharp differences between these results might be partially explained by different factors, including fetal circadian rhythms and pathological conditions [2, 3]. However, all studies included healthy fetuses and most had US scans performed during the afternoon [11, 12, 13, 14, 15, 18, 19]. Therefore, the inconsistencies shown in Table 1 suggest that the measures used in these studies lacked adequate reliability or validity.

Q2: The Authors did not mention the sample size calculation for their study. I would suggest reporting how the Authors chosen the number of included neonates.

**RESPONSE:** Because yawn is universally known as stereotyped behavioral pattern (see Table 1), we concluded that even a small sample (130 mouth openings) would be sufficient for our purposes, i.e. to assess the validity of the method proposed by Reissland and colleagues (in Study 1) and to test the feasibility of more sophisticated SVM-based methods for identify yawns (in Study 2). The results of Study 2 confirm this assumption; models highlighted good performances in distinguishing yawns and non-yawns. It is noteworthy that preterm neonates, despite representing a good model for fetal behavior, represent a very difficult population to sample and study. Because, in our experience, sample size calculation is generally not reported in studies adopting SVM or other kinds of classification algorithms, and classification performance is generally intended as the criterion for determining the required sample size for training (see https://www.ncbi.nlm.nih.gov/pmc/articles/PMC3307431/), we propose not to introduce this specification.

Q3: The authors have not adequately highlighted the strengths and limitations of their study. I suggest better specifying these points. The Authors use the Baby FACS coders as the gold standard for the evaluation of yawns, is it actually the gold standard? – The newborns yawns in air and fetus yawns in water, do Authors expect some changes?

**RESPONSE:** We fully embrace the reviewer's suggestions and inserted the pertinent considerations as a new paragraph in the discussion (lines 455-478, reported below).

“In order to establish this method as a viable option for identifying fetal yawns in clinical and research settings, additional work should be done to test it on fetal behavior. In particular, the suboptimal framing and variable quality that characterize ultrasonographic scans represent a ubiquitous issue in the study of fetal behavior. However, the fact that the proposed method only relies on the timing of mouth openings highlights a key advantage of this approach, that was specifically conceived in order to overcome the issues of identifying a complex facial behavior such as yawn from ultrasonographic scans. Another potential reason for concern regards the different conditions of fetuses compared with preterm neonates; for example, fetuses are immersed in amniotic fluid and the yawn of the near-term fetus might occasionally be mechanically constrained because of its position. However, there is unanimous agreement in the literature on the stability of the yawning behavioral pattern throughout life [12, 17, 21, 22, 23, 24]. Therefore, the advantages of using preterm neonates as a model for training classificatory algorithms far outweigh these potential issues.

A strength of the current study was the use of the SCPB [25], a rigorous coding system based on Baby FACS [22], to identify yawns. Because Baby FACS coding is based on multiple and redundant cues to each facial action, facial muscle actions can be reliably identified in fetuses [36], preterm and full-term neonates [37], and infants with facial anomalies [38] as well as typically developing infants. And because the basic coding units of Baby FACS are exhaustive and mutually exclusive, any complex facial movement can be precisely and unambiguously identified in terms of combinations and sequences of its constituent facial muscle actions. Therefore, Baby FACS, which has been referred to as the “gold standard” for coding infants’ facial expressions [39], is especially suited for coding yawning, sensory and perceptual responses [37], pain [40], and other fetal, neonatal and infant behaviors that don’t fit simplified templates for a limited set of emotional expressions.”

Q4: As appropriately stated by the authors, fetal yawning has been associated with proper neurological development. In this regard, the possibility to detect these fetal movements may play a pivotal importance as additional tool to assess fetal wellbeing in utero. I would suggest stressing the possible underlying causes of fetal neurological impairment, such as for example placental diseases (refer to: PMID: 28282763; PMID: 28466013; PMID: 28243732).

**RESPONSE:** We thank the reviewer for this important remark. References to this aspect were inserted at the beginning of the discussion and at the end of the discussion (respectively lines 414-417 and 493-496, reported below).

“The ability to identify yawns in fetuses and to distinguish yawns from non-yawn mouth openings is of interest because accurate detection of this widely observed behavior can have potential clinical importance for identifying early signs of neurodevelopmental abnormalities and other conditions such as placental diseases [35] in fetuses, very early preterm infants, and other populations at risk.”

“In conclusion, the development of a reliable and valid method for identifying yawning can provide a potentially valuable tool for studying perinatal behavior and for assessing fetal and preterm infant wellbeing. Future research using machine-learning systems could contribute to the development of sensitive measures for early diagnosis of neurological impairments and other disorders.”
