# Supplementary material for: Identifying fetal yawns based on temporal dynamics of mouth openings: A preterm neonate model using support vector machines (SVMs)
Source: PLoS One. 2019 Dec 19;14(12):e0226921. doi: 10.1371/journal.pone.0226921 (PMC6922391; doi:10.1371/journal.pone.0226921)
Supplement: Attachment — Submitted filename: Response to Reviewers.docx [file pone.0226921.s003.docx]

Reviewer #3: R3, Q3: I cannot agree that default values of SVM parameters ensure optimal classification results. In my opinion, for each of the considered models it is necessary to experimentally find C and γ values ensuring the best model performance. For example the
first 10 iterations of the hold-out validation can be used to find the parameters (from a reasonable range of C and γ) providing highest values of performance indices. Since it may be difficult to analyze several performance indicators simultaneously, F-Score (or similar) can be employed. However, even in this case the claim of the C and γ optimality is questionable.

**RESPONSE:** We really thank the reviewer for insisting on this important point. As suggested, we adopted grid search to optimize C and γ values (see lines 329-332, see below).

“Grid search based on 10-fold cross-validation error was employed to optimize the parameters C and gamma using the function “tune.svm”, within the interval [10^-3^, 10^3^]. Model A was optimized with C = 100 and gamma = 0.1 while Model B was optimized with C = 10 and gamma = 1.”

Moreover, because once we optimized these parameters within the interval [10^-3^, 10^3^] the full model (originally model E, now model A) highlighted the best performance (as is generally expected), we simplified the SVM analysis by deleting three models and retaining only the full model (Model A) and Model B (including the three-ways interaction of total duration, plateau duration and opening/closing asymmetry). The rationale for maintaining Model B is that it shows that the interaction of these three variables is responsible for a significant part of the ability to discriminate between yawns and non-yawn mouth openings. On the other hand, because the full model is now the best performing one and differences between models are smaller once C and gamma are optimized, we think that including all five original models would only contribute to make results less clear. Methods, results and discussion of Study 2 were modified accordingly.
